# Four hundred years of cork imaging: New advances in the characterization of the cork structure

**DOI:** 10.1038/s41598-019-55193-9

**Published:** 2019-12-23

**Authors:** Kevin Crouvisier-Urion, Julie Chanut, Aurélie Lagorce, Pascale Winckler, Zi Wang, Pieter Verboven, Bart Nicolai, Jeannine Lherminier, Eric Ferret, Régis D. Gougeon, Jean-Pierre Bellat, Thomas Karbowiak

**Affiliations:** 1 Univ. Bourgogne Franche-Comté, Agrosup Dijon, UMR PAM A 02.102, 1 Esplanade Erasme, 21000 Dijon, France; 2 Univ. Bourgogne Franche-Comté, ICB-UMR 6303 CNRS, 9 avenue Alain Savary, B.P. 47870, 21078 Dijon, France; 3 KU Leuven – University of Leuven, Biosyst-MeBioS, W. de Croylaan 42, B-3001 Heverlee, Belgium; 4 Agroécologie, Agrosup Dijon, CNRS, INRA, Univ. Bourgogne Franche-Comté, 21000 Dijon, France; 5 Univ. Bourgogne Franche-Comté, Inst Univ Vigne & Vin, 1 Rue Claude Ladrey, 21078 Dijon, France; 6 Dimacell imaging facility, Agrosup Dijon, INRA, INSERM, Univ. Bourgogne Franche-Comté, 21000 Dijon, France

**Keywords:** Plant sciences, Biomaterials - cells

## Abstract

In 1665, Robert Hooke was the first to observe cork cells and their characteristic hexagonal shape, using the first optical microscope, which was invented by him at that time. With the evolution of imaging techniques, the structure of cork has been analysed with greater accuracy over time. This work presents the latest advances in the characterization of this unique material through a multiscale approach. Such investigation brings new insight into the architecture of cork, particularly the differences between the cells of the phellem and those bordering the lenticels. In the latter case, cell differentiation from the lenticular phellogen was restricted to one cell layer, which leads to a cell wall that is 10 times thicker for lenticels. They also displayed a different chemical composition because of unsuberization and a high lignin content in lenticels. Such advances in the knowledge of the structure and composition of cork cells contributes to a better understanding of the macroporosity of cork, down to the nanoscale.

## Introduction

**Figure 1:**
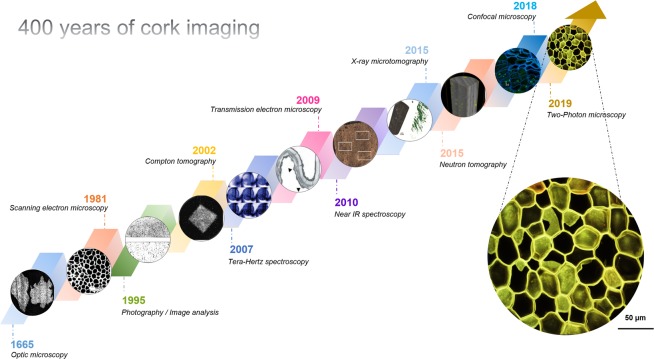
Overview of 400 years of cork imaging.

**Figure 2:**
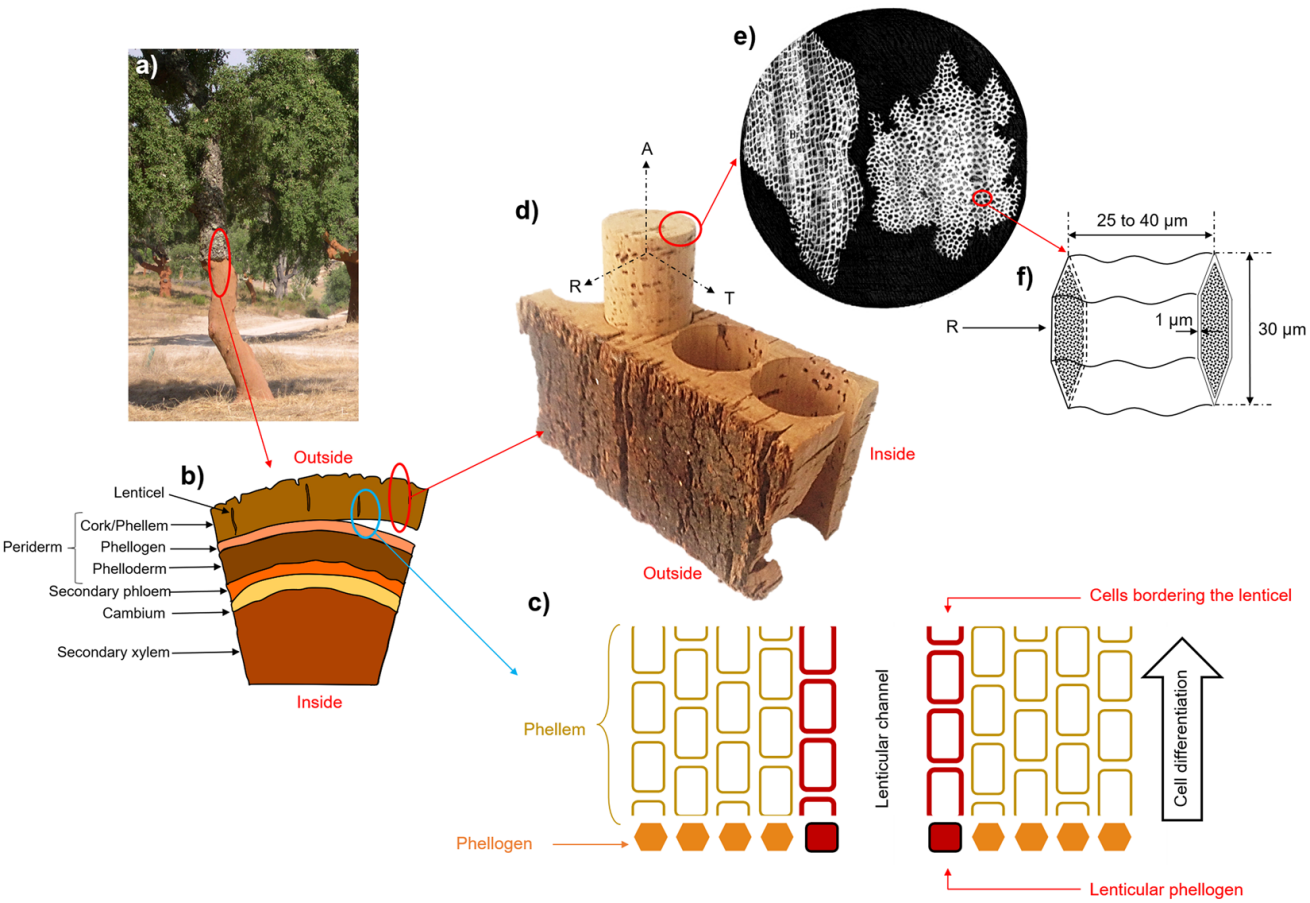
(**a**) *Quercus suber L*. tree after cork bark harvesting. (**b**) Representation of the transverse section of cork tree. (**c**) Zoom on the phellogen region with cellular differentiation. (**d**) Tubbing of cork stopper from the cork bark. Letters A, R and T refers to as the axial, radial and tangential directions, respectively. (**e**) First observation of cork cells by Robert Hooke in 1665^1^. (**f**) Characteristic shape and dimensions of a phellem cell.

Robert Hooke, inventor of the first microscope, published a collection of his hand-drawn observations of biological samples in 1665 in a book entitled *Micrographia*^1^ (Fig. 1). Among the studies of different plants or animals by Hooke, one of the most famous remains the first observation of cork cells from *Quercus suber* (Fig. 2e). Optical microscopy allowed this first representation of the basic biological unit by Hooke, which was then defined as a “cell”^2^. Cork was described as an alveolar material composed of dead and empty closed cells. It corresponds to the bulk of cork tissue including lenticels, which are open macroporous channels. Cork tissue is referred to as the phellem in the present work. A finer description was only reported in 1981 by Gibson *et al*.^3^ using scanning electron microscopy (SEM). While Hooke only reported the orientation of cork cells in two directions, Gibson highlighted their specific 3D structure, which is at the origin of the anisotropy of the material. In fact, the structure of phellem cells varies according to the observation plane (Fig. 2f). In the plane perpendicular to the radial direction, cells appear as hexagons that are arranged according to a honeycomb structure. In the two other planes, either perpendicular to the tangential or to the axial directions, they exhibit a rectangular shape and are stacked up, similar to a brick wall. Furthermore, in the plane perpendicular to the tangential direction, the cell walls are corrugated. Gibson was also the first to point out the relationship between the cork cell structure and the mechanical properties of the material^3,4^. Its remarkable near-zero Poisson’s ratio along the axial direction still makes cork an unique reference for the design of new advanced materials^5^. A model of the phellem architecture^6^, as an alveolar system composed of closed porosity, and with characteristic dimensions, was proposed based on SEM observations^3,7^. However, no 3D imaging of cork cells was available at that time

.

The typical anisotropic structure of phellem cells is attributed to the biological development of *Quercus suber* L. tree. Phellem results from the cell differentiation of the phellogen, which is restricted to one cell layer (Fig. 2b). After the division from the phellogen cell, a limited thickening of the primary cell wall, which is rich in lignin, occurs. Phellem cells are then subjected to a very rapid suberization as the consequence of an important metabolic activity with an increase in smooth endoplasmic reticulum and the production of vesicles by dictyosomes^8^. The genes that are responsible for this metabolic activity have been identified and are known to lead to the synthesis and linkages of the aromatic and aliphatic precursors of suberin^9^. Thus, the thick secondary phellem cell wall is a result of this fast suberization. During this phenomenon, the protoplasmic layer also tends to be detached from the cell wall. Suberin deposition is immediately followed by the formation of the tertiary wall, which is mainly composed of polysaccharides and extractable compounds. Therefore, the development of phellem cells gives rise to a cell wall that is divided into three layers, which are mainly composed of suberin (45 wt %), lignin (22 wt %), polysaccharides (18 wt %) and extractable compounds, such as lipids^10^, terpenoids and phenolic compounds (15 wt %)^11,12^. Crossing the cell walls, thin channels with a diameter of ∼50 nm, called plasmodesmata, were recently identified using transmission electron microscopy (TEM)^13^. Plasmodesmata act as intercellular channels that mediate the cell-to-cell transport of signaling molecules, such as non-cell autonomous proteins or RNAs, in the living cells^14^. After their development, cork cells are autolyzed and undergo programmed cell death in response to environmental cues^15,16^. Thus, the resulting layer of phellem, which is composed of dead cells, is an efficient insulator, either acting as a barrier to dehydration or as protection against fire for the oak tree in the Mediterranean peninsula.

Nevertheless, to ensure the gaseous exchanges between the oak tree and the environment, the phellem is sprinkled with lenticular phellogen (higher meristematic activity), which leads to the formation of lenticels^17,18^ (Fig. 2c). Lenticels are channels with a diameter in the millimeter scale that represent an open macroporosity across the cork bark. In the cork industry, cork stoppers are punched out from the tree bark perpendicularly to lenticels (Fig. 2a,b,d). The quality and price of natural cork stoppers are defined according to the external apparent surface porosity constituted by these lenticels, which is often visually determined by qualified workers. However, imaging coupled with image analysis is being used more frequently in this setting to classify cork quality. In that case, the sorting of cork is based on the surface density of lenticels, as determined by the 2D analysis of photographs^19,20^. This sorting is sometimes also performed by probing the inner structure of the cork using X-ray radiography^19^. A higher number of lenticels indicates a lower quality of the cork. Several other imaging techniques have been applied to study cork, such as terahertz millimeter wave^21^, near-infrared spectroscopies^22^ and more recently confocal microscopy^16^ (Fig. 1) X-ray^23,24^ and neutron tomographies^19^ were also used, showing that lenticels are not interconnected within a cork stopper. This implies that the limiting step in gas transfer is the crossing of cell walls. However, the mechanism of diffusion through the cork cell wall has not been fully identified^25^, as it can occur via surface diffusion through the polymers composing the cell wall^26,27^ and/or via Knudsen diffusion through the plasmodesmata^28,29^. The use of more recent and powerful imaging techniques can be an effective mean to investigate further the cell wall structure of cork.

From the invention of the first microscope to the present day, imaging has been used to describe the structure of cork, the arrangement of its cells and their characteristic dimensions and the spatial distribution of lenticels. However, the information available in the literature concerning the differences between the phellem cells and the lenticel cells in terms of structure and composition is scarce. The objective of this paper was to investigate this aspect based on multiscale imaging. First, at the mesoscale, X-ray computed tomography was applied to characterize the structure and porosity of solid cork stoppers. Second, structural differences between the cells composing the phellem and those bordering the lenticels were highlighted based on the use of two-photon microscopy. Finally, at the nanoscale, TEM was performed to observe the structure of the plasmodesmata that cross the cell walls.

## Results and Discussion

### Structural differences related to cork quality

**Figure 3:**
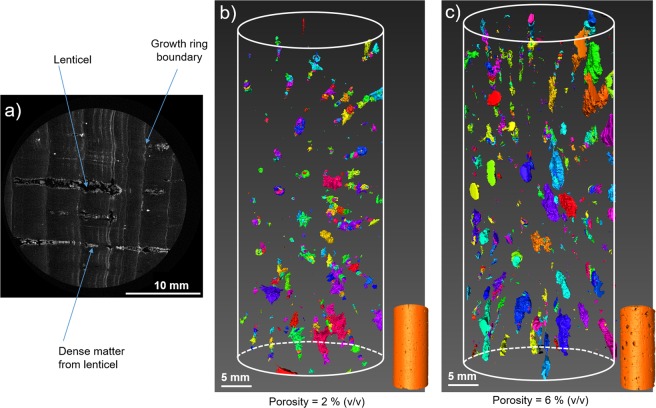
(**a**) Cross section of a cork stopper, from X-ray tomography, illustrating the lenticels as holes (black pixels) with densified matter all-around (white pixels). Thresholding on black pixels was applied to determine the porosity of full cork stoppers of (**b**) high-quality and (**c**) low-quality grade.

Lenticel content is the main criterion defining cork quality. The quantification of lenticel density is made visually by qualified workers or by 2D imaging. However, this does not allow the characterization of the internal porosity of cork stoppers, as the sorting is based exclusively on the surface density of lenticels. To assess the inner porosity that is attributed to lenticels, X-ray tomography was performed on high-quality and low-quality cork stoppers. Figure 3 displays the results obtained upon image reconstruction. The observation of the cork stopper in the plane perpendicular to the axial direction (Fig. 3a) showed that empty tube shapes, corresponding to lenticels, are surrounded by dense matter. Some growth rings boundary that crossed lenticels perpendicularly, also appeared as dense matter (Fig. 3a).

First, the porous volume of lenticels was evaluated using thresholding on black pixels on each slice of the stopper. The same image treatment was applied to achieve a 3D representation of the macroporous network of lenticels within the reconstructed cork stoppers of high (Fig. 3b) or low (Fig. 3c) quality. In both cases, lenticels were not interconnected within the stoppers. Moreover, a significant difference in porous volume was noticeable: ~2% (v/v) for the higher grade and ~6% (v/v) for the lower grade stoppers. A few studies based on neutron or X-ray tomography have reported porous volumes ranging from 2.0 to 5.9% (v/v) for high-quality stoppers and from 2.6 to 20.5% (v/v) for low-quality stoppers^19,24^. Despite the heterogeneity of this natural material, such variability is also dependent on the thresholding. Nevertheless, the internal porosity values determined here remain in accordance with the sorting that is performed in this industry, which is based on surface porosity^19^.

Second, the proportion of dense matter surrounding lenticels was calculated considering white pixels but without taking into account growth rings boundary. The volume fraction of this dense matter bordering lenticels is more important for the lower grade (2.5%) than for the higher grade (1.2%) stoppers. On the one hand, it has been shown that a higher amount of lenticels increases the rigidity of the material^18,30,31^. Thus, such increase is attributed to the densification of lenticels, which rigidifies the cork structure. Unfortunately, on the other hand, no clear relationship between the presence of lenticels and the permeability of cork to gases has been established^24^. A better characterization of their structure at a lower scale will certainly help to understand better the role played by these densified pores in the gas barrier properties of cork.

X-ray tomography is a high-performance technique that can be used to investigate the inner structure of cork stoppers in a non-destructive way. Although it is time consuming when high resolution is required, it is noteworthy that there has been progress in X-ray tomography toward faster and in-line applications^32^. Even if the cell structure can also be assessed using this technique (limited to the hundred micrometer scale), other imaging techniques are used to study more accurately the cells that compose the phellem and the lenticels.

### Phellem and lenticels: investigation of cell structure

**Figure 4:**
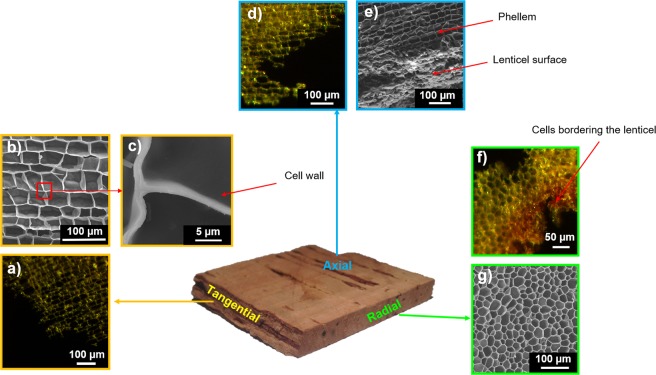
Cork structure observed from optical microscopy (OM) and scanning electron microscopy (SEM). View from the: **Tangential direction** (or radial plane). (**a**) Phellem, from OM. **b)** Phellem, from SEM. (**c)** Zoom in the cork cell wall. **Axial direction** (or transverse plane). (**d)** Phellem, from OM. (**e)** Phellem and lenticel surface, from SEM. **Radial direction** (or tangential plane). (**f)** Phellem and lenticel, from OM. (**g**) Phellem, from SEM.

It is preferable to observe cork cells via optical microscopy using very thin cork samples (with a thickness close to the size of a cell) to distinguish the cellular pattern clearly. Using this experimental precaution, the specific geometry of cork cells can be discerned: a hexagonal shape along the plane perpendicular to the radial direction and a rectangular shape along the planes perpendicular to the tangential and axial directions (Fig. 4). The cells that border the lenticels seem to be densified (Fig. 4f), as observed previously using X-ray tomography.

The observation of cork cells is largely improved by the use of SEM. It then becomes possible to determine better the geometry of cells and their dimensions, with a resolution lower than the micrometer, which approximately corresponds to the thickness of the cell wall^33^. Furthermore, the development of image analysis allowed a more accurate description of the geometry of phellem cells. It is worth noting that the geometry of phellem cells is not a perfect hexagon^34^ in the plane perpendicular to the radial direction; rather, deformed hexagons or pentagons are observed^7^ (Fig. 4f,g). In the planes perpendicular to the axial and tangential directions, the phellem displays a brick-wall pattern, with larger cells in the tangential direction (~40 µm in size) compared with the axial direction. The geometry of the cells that comprise lenticels was not regular, and they appeared to be densified (Fig. 4e,f).

Even if SEM is a powerful tool for the analysis of the structure of cork cells, observations are performed on samples coated with carbon or gold and maintained under vacuum. These experimental conditions might alter the surface by masking defects because of the coating or by modifying the material, which undergoes dehydration during image acquisition. Furthermore, although the resolution of SEM is rather good, better than 1 µm, it was interesting to shift toward a method that allows the 3D observation of cork cells and provides more information about cork structure and cell wall composition in environmental conditions.

**Figure 5:**
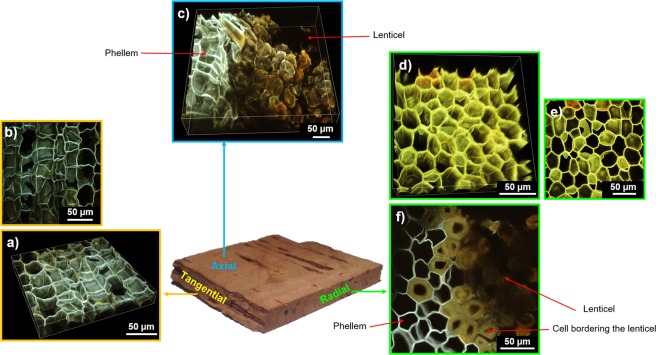
Cork structure observed from two-photon microscopy. View from the: **Tangential direction** (or radial plane). (**a**) 3D representation of phellem. (**b**) Maximum intensity projection stack obtained from the previous 3D representation. **Axial direction** (or transverse plane). (**c**) 3D representation of phellem including a lenticel. **Radial direction** (or tangential plane). (**d**) 3D representation of phellem. (**e**) Maximum intensity projection obtained from the previous 3D representation. (**f**) 2D representation of phellem including a lenticel and highlighting the densification in cells bordering the lenticel.

As confocal microscopy, two-photon microscopy is commonly used to perform 3D representations of plant or animal tissues^35^. In plants, these techniques allow to observe the fluorescence provided by a previous labelling step or may be based on cell autofluorescence, which is usually quite strong. Lignin and, to a lesser extent, suberin, are two natural fluorophores, that display a range of emission from 440 to 540 nm and from 460 to 500 nm, respectively^36,37^. They provide a natural autofluorescence to the cork cell wall that can easily be observed by confocal or two-photon microscopy. These imaging methods are particularly interesting because they can provide not only 2D images of cork cells^38^, but also 3D representations, without any previous labelling or vacuum treatment. However, 3D imaging of plant tissue remains challenging because of the strong absorption of the excitation beam along the depth of the sample. As two-photon absorption mainly occurs in the focal plane, the excitation beam is weakly absorbed in the out of focus planes. Thus, it allows a better penetration depth in thick and opaque samples, as cork cells. This technique enabled us to obtain a 3D map of the top cork cell layers (Fig. 5a,c,d). The two-photon microscope is designed for hydrated samples imaging. Unfortunately, because of the alveolar structure of the material (∼80% of the material is filled with air), it was not possible to penetrate deeply enough into the dry material without optical aberrations. Cork sample was thus previously immersed in water in order to fill cork cells, which largely improved image acquisition. The most important contribution of this technique was a better understanding of the structural differences between cells from the phellem and from lenticels.

First, in the plane perpendicular to radial direction, cells at the phellem/lenticel boundary had a thicker wall. They displayed a characteristic thickness of around 10 µm, compared with 1 µm for the phellem cell wall (Fig. 5f). In addition, it is noteworthy that this cell differentiation in cork was restricted to one cell layer. This observation is in accordance with the previous X-ray tomography reconstruction showing densified matter at the edge of the lenticels (Figs.3a and 5f). Moreover, the fluorescence emission was different between these densified cell walls bordering lenticels and those from the phellem. This might be attributed to a different chemical composition^39^. To analyse this phenomenon further, imaging was coupled with a chemical composition analysis of the cell wall using X-ray Photoelectron Spectroscopy (XPS).

XPS analysis clearly revealed a significant difference between the phellem and the lenticels (Table 1). The cell walls bordering lenticels displayed a higher carbon content (C/O ratio of 4.28 ± 0.16) compared with those from the phellem (C/O ratio of 3.79 ± 0.09). This indicated a change in the chemical composition caused by cell differentiation. Regarding the major components of the cork cell wall (45 wt% of suberin and 22 wt% of lignin), it was noticeable that these compounds also presented a difference in their C/O ratio (3.29 and 4.5 for suberin and lignin, respectively)^18,40^. The comparison of the C/O ratios obtained by XPS for the phellem and lenticels with those of lignin and suberin revealed that the cells bordering lenticels were mostly densified with lignin, while phellem cells were mainly composed of suberin. This is also in complete agreement with the cork histological development. Lenticular phellogen differentiation leads to the development of unsuberized cells, which are mainly composed of lignin^18,41^.

**Table 1:** C/O ratio determined on cork phellem and lenticel from XPS analysis (n = 4), and compared to C/O ratio, from literature, of lignin and suberin extracted from cork.

Cork region or component analysed	C/O ratio	References
Phellem	3.79 ± 0.09^a^	Present work
Lenticel	4.28 ± 0.16^b^	Present work
Suberin	3.29	*Cordeiro et al*.^40^
Lignin	4.50	*Pereira et al*.^17^

Superscript letters indicate significant difference for C/O ratio between phellem and lenticel (Student test, p < 0.05).

In conclusion, the phellem and the lenticels did not exhibit a similar structure or chemical composition. Lignification occurred in the cells located at the periphery of lenticular channels. This phenomenon only concerned one cell layer. From a mechanical point of view, this confers more rigidity to the material. Regarding gas transfer, lenticels could, at first glance, be considered as macropores, in which Darcy’s diffusion can take place. Nevertheless, as they are not interconnected in the axial direction, the limiting step in gas transfer within a cork stopper is the diffusion at the cellular scale through the cell wall. Therefore, as lenticels are composed of cells with thicker cell walls, these thicker structures might also be regarded as a better barrier to gas transfer in cork stoppers.

### Phellem cell wall at the nanoscale

**Figure 6:**
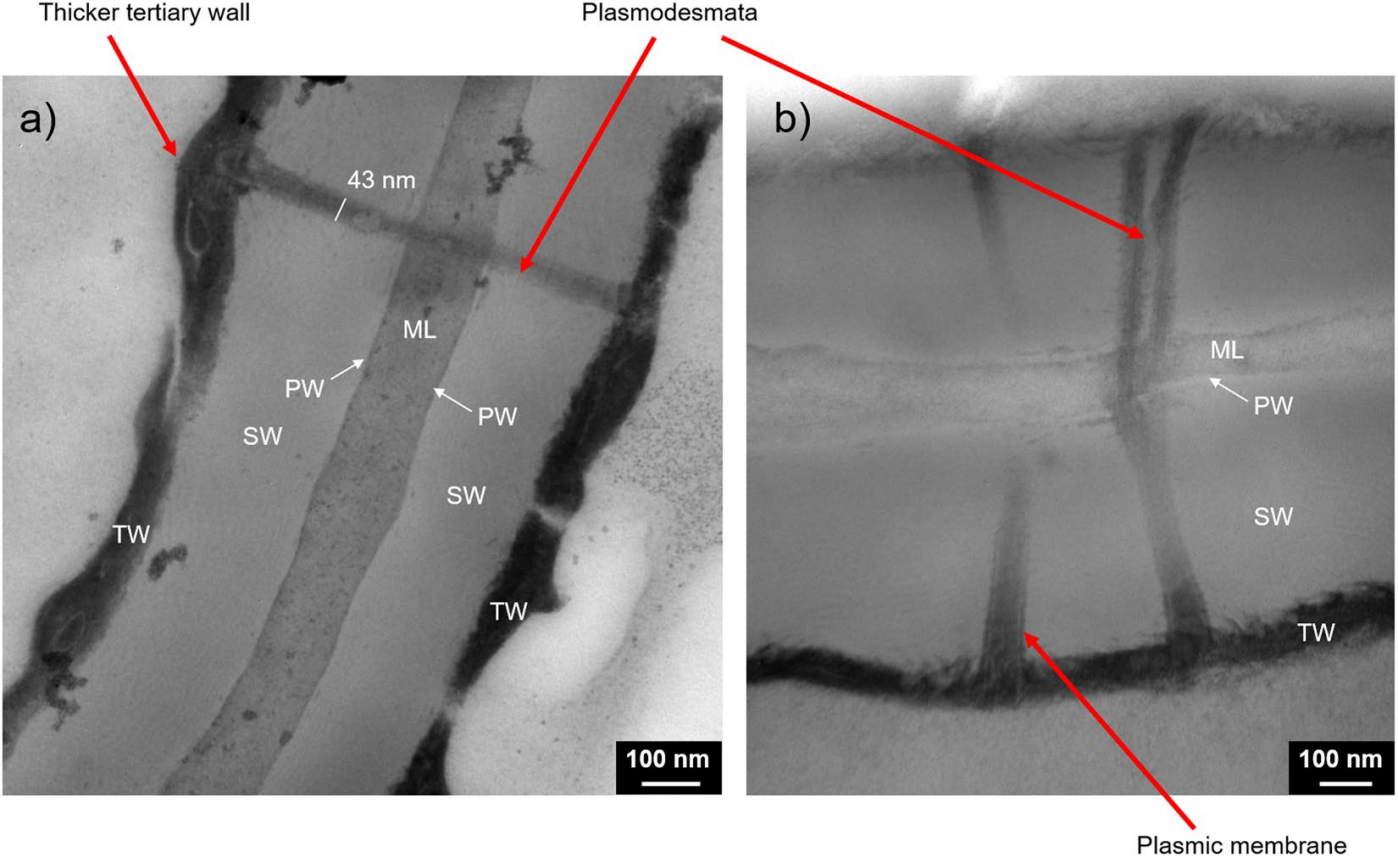
Phellem cell walls observed from Transmission Electron Microscopy (TEM). The cell wall is composed of the four successive layers: Middle Lamella (ML), Primary Wall (PW), Secondary Wall (SW) and Tertiary Wall (TW). The selected TEM pictures highlight a plasmodesmata crossing the cell wall. (**a**) A thicker region of the tertiary wall can be seen at the extremities of the plasmodesmata. (**b**) The plasmic membrane is visible inside the plasmodesmata.

The mechanisms underlying the diffusion of gas across cork cells remains mostly unknown, in particular because of the incomplete knowledge of the structure of the plasmodesmata that cross phellem cell walls. On the one hand, functional plasmodesmata (as in living plants cells) with a diameter of ∼50 nm would allow gas exchange via Knudsen diffusion. On the other hand, densified plasmodesmata (after cell death) would constitute a barrier to gas transfer, which implies that gas diffusion would be governed by surface diffusion through the cell walls. Therefore, the structure of plasmodesmata is a key point in the understanding of the phenomenon of molecular diffusion in cork. TEM appears to be a tool of interest for studying such structure at the nanoscale. Figure 6 displays TEM observations of the phellem cell wall.

First, it was noticeable that plasmodesmata crossed the four layers that constitute the phellem cell wall, namely the middle lamella, the primary wall, the secondary wall and the tertiary wall, as shown in Fig. 6. The structure of these layers is in agreement with previous descriptions^18,42^. It was difficult to distinguish the primary wall, which is very thin, at the periphery of the middle lamella. Moreover, both the structure and the chemical composition of these constituting layers appeared to be different, considering the range of contrasts obtained from TEM (Fig. 6). Even if the polymers composing the cell walls are interconnected, it has been shown that lignin is mainly located in the middle lamella, whereas suberin is mostly present in the secondary and tertiary walls^18^.

Regarding the shape and structure of plasmodesmata, they were mostly unbranched, with a diameter of 50 ± 10 nm, and were not distributed uniformly along the phellem cell wall. Plasmodesmata were also sometimes branched, with characteristic V-shaped channels (Fig. 6b). Furthermore, although cork cells are a dead material, the residual plasmic membrane was preserved and was visible at the periphery of plasmodesmata (Fig. 6b). Plasmodesmata, which allow exchanges between cells in the living tissue, appeared, in the phellem, obstructed at their extremities by the tertiary wall. In some cases, a deposition of dense cytoplasmic residues was observed, in addition to the tertiary wall, which appear thicker at the end of plasmodesmata and desmotubules (Fig. 6a). Such matter deposition at the ends of plasmodesmata is a well-known mechanism in plant cells after a stressor, as it regulates the diffusion of metabolites between living cells or is related to programmed cell death^43,44^. Such plasmodesmata obstruction was previously observed in cork cells^13^. However, even if plasmodesmata densification has been noted in other suberized plants, such as potatoes^45^, the obstruction of these nanochannels seems to be specific to cork. The loss of cytoplasm after the programmed cell death leads to the rapid suberization of the phellem^46^, which is followed by the obstruction of plasmodesmata in the case of cork.

## Conclusions

In conclusion, a multiscale analysis of the cork structure by coupling different imaging techniques was performed. X-ray tomography was well suited for the analysis of the macroporosity of cork stoppers and for determining the volume fraction of lenticels within the material. The apparent quality of cork, as estimated from the density of lenticels from the outer surface, was fairly representative of the inner porosity of the cork. In addition, X-ray tomography also showed a material densification at the boundary of lenticels. In the near future, X-ray computed tomography would probably allow to investigate the 3D structure of cork at the cellular scale.

The characterization of the cell structure of cork was first achieved using optical microscopy and SEM. To deepen the understanding of the densification phenomenon, a more in-depth study performed at the cellular scale using two-photon microscopy revealed differences in structure and chemical composition between the cells of the phellem and those of lenticels. Cells of lenticels exhibited a cell wall that was 10 times thicker than that of the phellem cells. This cell differentiation occurred over one cell layer. Based on XPS measurements, it appeared that such thicker cell walls at the lenticel boundary were mainly composed of lignin, which originates from lenticular phellogen differentiation, while the cell walls of the phellem were mostly composed of suberin. To go further, it would be interesting to perform specific labelling to be able to identify the different polymers and their positions inside the cell wall.

At the nanoscale, the structural characteristics of the plasmodesmata that cross the cell walls were investigated by TEM. Plasmodesmata, which are open nanometric channels that allow exchanges between living cells, underwent a phenomenon of suberization and seemed to be obstructed after programmed cell death during the development of the cork bark. The use of TEM tomography could further help reveal the plasmodesmata ultrastructure with better 3D resolution.

Therefore, the multiscale imaging approach reported here brought new insights into the characterization of the cork structure. This provides the basis for the further development of more accurate structural models to establish relationships with the functional properties of the material.

## Material and Methods

### Cork stoppers

Raw natural cork stoppers from *Quercus suber* were supplied from Bouchons Trescases S.A. (France). All experiments were performed on high-quality cork stoppers (class 0; diameter, 24 mm; length, 49 mm) without any washing or surface treatment. Lower-quality cork stopper (class 4; diameter, 24 mm; length, 49 mm) was also used for X-ray tomography.

## Imaging

### Optical microscopy

Optical microscopy images of thin cork slices were obtained in reflexion mode using a Nikon TE 2000 microscope equipped with a Plan Fluor 40x objective (NA, 0.6; Nikon) and a DS-2M colour camera (Nikon). Image acquisition was performed in three observation planes.

### Scanning electron microscopy

Cork cells were observed by SEM using a Jeol JSM 7600 F microscope (15 kV). Prior to imaging, cork samples were cut with a razor blade, and coated with carbon (15–20 nm). Image acquisition was performed in three observation planes.

### Transmission electron microscopy

Cork samples were high-pressure frozen in a Leica HPM 100 high-pressure freezer. Freeze substitution was performed subsequently using a Leica EM AFS 2 apparatus. Samples were substituted at −90 °C for 48 h with 2% osmium tetroxide in acetone. The temperature was gradually increased to −60 °C for 46 h, then to −30 °C for 10 h using the same fluid (slope, 3 °C.h^−1^). Samples were rinsed with pure acetone at −30 °C, and then gradually infiltrated with mixtures of acetone/Epon resin, with increasing concentration of resin and temperature, up to 20 °C. Polymerization was performed at 60 °C. The blocks of Epon-embedded cork samples were thin-sectioned, and sections were collected onto carbon/collodion-coated copper grids. Grids were stained with UranyLess for 8 min and lead citrate for 2 min (EMS). Sections were observed on a Hitachi H7500 transmission electron microscope operating at 80 kV and equipped with an AMT camera driven by the AMT software (Hitachi). Five samples were frozen, and three observations were performed on these samples at several levels.

### Two-photon microscopy

Two-photon imaging microscopy was performed to achieve 3D representations of the cell structure of cork based on lignin and suberin autofluorescence^37^. Images were collected on a Nikon A1-MP scanning microscope equipped with a Plan APO IR 60x objective (NA, 1.27; Water Immersion, Nikon) at a scanning speed of 1 frame.s^−1^. An IR laser (Chameleon, Coherent) was used to provide excitation at 800 nm. The autofluorescence emission of cork was collected on four detection channels: FF01-492/SP (400–492 nm), FF03-525/50 (500–550 nm), FF01-575/25 (563–588 nm) and FF01-629/56 (601–657 nm) (Semrock). Images provided in the manuscript were obtained by merging these four detection channels without any other spectral selection.

Prior to observation, cork was immersed in distilled water at 25 °C, for 1 month to reach a high level of humidity, which improves image acquisition (as the objective used is not designed for dry samples). The 3D representation was obtained by superimposing of images scanned along the depth of the samples (140 µm) with 0.5 µm acquisition step. Observations were performed along the plane perpendicular to the radial and axial directions of cork. Images and reconstructions presented in this work were obtained from the same cork sample.

### X-ray tomography

High- and low-quality cork samples were subject to micro-computed tomography scans using a Phoenix Nanotom micro-CT system (General Electric). The X-ray tube was tuned down to 45 kV and 445 µA to accommodate the low density of the material. As the height of the cork samples exceeded the height of the detector, the scans were completed in a series by scanning 1/3 of the cork at a time (heightwise). Projection images (2400) were captured with an exposure time of 500 ms over a 360° rotation with a 12 µm pixel resolution, resulting in a total acquisition time of 20 min per 1/3 of sample (and 1 h per whole cork sample). Octopus Reconstruction 8.9.2 (XRE) was used to produce reconstruction images via the filtered back projection algorithm, with the ring artefact correction and noise suppression modules enabled. Reconstructed images were then down sampled to 8 bits to reduce computational requirements during processing and analysis.

Reconstructed images were imported into Avizo for stitching and image processing. Each of the three image stacks obtained per cork sample was first trimmed and then subsequently registered to align the stacks for resampling/stitching. The stitched and therefore completely reconstructed cork samples were then subjected to further analysis.

Reconstructed 3D objects were then analysed using Avizo software (Thermo Fischer Scientific). As cork is composed of atoms with a similar X-ray absorption factor, the very dense part of the matter inside the material will absorb a large amount of X-rays and appear in white on the reconstruction. By contrast, the pores will appear in black. Based on these considerations, it was possible to analyse the images obtained from stoppers. Each voxel in this 3D representation had a specific grey value (on a scale from 0 to 250), on which thresholding was based. Two thresholding were applied to isolate the lenticels (from 1 to 10 on the greyscale) and the densified matter (from 130 to 250 on the greyscale). The number of voxels corresponding to these two distinct thresholding steps was then determined to calculate the volume occupied by the pores and by the densified matter, respectively. It was also possible to represent the voxels associated with each thresholding within the full cork stopper and to observe their interconnection (interconnected voxels appear in the same colour in Fig. 3). One stopper for each quality was analysed.

### X-ray photoelectron spectroscopy (XPS)

For XPS analysis, cork was used as a powder and collected separately from the phellem and from the lenticels by scratching the surface with a scalpel, paying particular attention to only collect the cell surface. It was sieved to obtain a powder granulometry <50 µm (corresponding to the size of a single cork cell) to determine its chemical composition.

XPS analysis was performed using a PHI Versaprobe 5000 apparatus. A monochromatic 50 W Al Kα radiation (1486.7 eV) was used as the X-ray source. Measurements were carried out at room temperature inside an ultrahigh vacuum compartment. A spot with a diameter of ∼200 µm was analysed. High-resolution (pass energy, 58 eV) 45° emission angle integrated scans were acquired. XPS spectra were normalized according to C-C/C-H bonds (carbon 1 s line at 284.6 eV). A quantification aimed at determining the percentage of carbon, oxygen and nitrogen was carried out on four replicates. Statistical analysis was performed to compare the two sets of samples using the Student’s test (p < 0.05).

## Data Availability

The authors can confirm that all relevant data are included in the paper
